# Edge intelligence for poultry welfare: Utilizing tiny machine learning neural network processors for vocalization analysis

**DOI:** 10.1371/journal.pone.0316920

**Published:** 2025-01-16

**Authors:** Ramasamy Srinivasagan, Mohammed Shawky El Sayed, Mohammed Ibrahim Al-Rasheed, Ali Saeed Alzahrani

**Affiliations:** 1 Computer Engineering, CCSIT, King Faisal University, Al Hufuf, Kingdom of Saudi Arabia; 2 Avian Research Center, King Faisal University, Al-Ahsa, Saudi Arabia; 3 College of Veterinary Medicine, King Faisal University, Al-Ahsa, Saudi Arabia; Ain Shams University Faculty of Agriculture, EGYPT

## Abstract

The health of poultry flock is crucial in sustainable farming. Recent advances in machine learning and speech analysis have opened up opportunities for real-time monitoring of the behavior and health of flock. However, there has been little research on using Tiny Machine Learning (Tiny ML) for continuous vocalization monitoring in poultry. This study addresses this gap by developing and deploying Tiny ML models on low-power edge devices to monitor chicken vocalizations. The focus is on overcoming challenges such as memory limitations, processing power, and battery life to ensure practical implementation in agricultural settings. In collaboration with avian researchers, a diverse dataset of poultry vocalizations representing a range of health and environmental conditions was created to train and validate the algorithms. Digital Signal Processing (DSP) blocks of the Edge Impulse platform were used to generate spectral features for studying fowl vocalization. A one-dimensional Convolutional Neural Network (CNN) model was employed for classification. The study emphasizes accurately identifying and categorizing different chicken noises associated with emotional states such as discomfort, hunger, and satisfaction. To improve accuracy and reduce background noise, noise-robust Tiny ML algorithms were developed. Before the removal of background noise, our average accuracy and F1 scores were 91.6% and 0.92, respectively. After the removal, they improved to 96.6% and 0.95.

## I Introduction and background

The health and welfare of poultry flocks play an essential role in sustainable farming practices. Monitoring poultry in real-time offers potential benefits for farm management, improving both productivity and animal welfare. Traditional monitoring approaches, however, tend to be labor-intensive and inconsistent, with limited capability for continuous supervision. Emerging machine learning techniques, especially in vocalization analysis, offer new possibilities for real-time poultry health and behavior assessment, potentially transforming the way flocks are managed on a daily basis.

The digital revolution in animal health is reshaping traditional approaches to disease prevention and management, with Artificial Intelligence (AI) playing a pivotal role in proactive intervention. Governments are encouraged to support this transformation through adaptive legislation and enhanced rural connectivity [1]. The U.S. AI animal health market is projected to grow significantly, with the global industry expected to expand from USD 54 billion in 2022 to USD 85.07 billion by 2032. Notably, the Asia-Pacific region is anticipated to see the highest growth rate [2]. Efforts by the Environmental Protection Agency (EPA) to update effluent limit guidelines (ELGs) reflect an evolving regulatory focus on balancing production with environmental sustainability [3]. Innovations like Internet of Things (IoT), Augmented Reality (AR), extended Reality(XR) help poultry farmers monitor and predict key factors such as feed, water, and climate issues, showcasing the potential of AI-driven solutions [4,5]. Various studies have emphasized the potential of deep learning for poultry health, and achieved better accuracy in classifying chicken growth, for behaviors and welfare analysis using You Only Look Once (YOLOv5), Convolutional Neural Network (CNN), Precision Live Stock Farming [6–13]. The role of acoustics in poultry monitoring has been widely studied and demonstrated how vocalization frequency correlates with stress and age, using acoustic signal processing and machine learning in welfare assessment [14–18]. The studies conducted using wavelet entropy and Mel frequency cepstral coefficients (MFCC) led to higher accuracy in classification [19]. IoT-based frameworks integrate ML for real-time disease prediction [20]. The light-VGG11 (Visual Geometry Group) model was proposed for efficient distress vocalization detection [21]. Recent studies explored stress-induced vocalizations, advocating for non-invasive welfare monitoring [22,23]. Machine Learning for vocal and behavioral analysis, reported high accuracy in classification [24,25]. Significant contributions in data processing for poultry monitoring emphasizing scalable, edge-computing solutions were reported in [26–29]. Optimized sound feature extraction for broiler sound classification reaching near-perfect accuracy was reported in [30].

All the above studies have explored the application of machine learning in animal sound analysis to detect states of health or distress. For instance, convolutional neural networks (CNNs) and other deep learning models have demonstrated high accuracy in identifying specific animal sounds associated with particular conditions or behaviors. However, these approaches are commonly implemented on high-power computers.

Platforms like Edge Impulse have simplified Tiny ML deployment for low-power devices, as highlighted in [31,32]. These platforms and domain-specific applications such as shelf-life estimation using Tiny ML underline the relevance of lightweight models in agriculture [33]. Tiny ML is an ideal choice for agricultural settings as it provides real-time data analysis directly at the source, enabling autonomous monitoring without reliance on high-power infrastructure.

History of AI development related to this study:

1980s: Early research focused on understanding animal behavior through acoustic signals, particularly in avian species. Spectrograms were used to visualize sound frequencies, aiding manual classification [34].
1990s: Advances in signal processing, such as Fourier Transforms, enabled researchers to extract basic sound features. These methods laid the groundwork for automated animal sound classification systems but were largely limited to small datasets and lacked real-time capability [35].
2000s: Machine Learning Techniques and algorithms like SVM and KNN were first used to classify animal vocalizations based on extracted features such as Mel Frequency Cepstral Coefficients (MFCCs). Hidden Markov Models (HMMs) were employed for sequential data analysis, allowing researchers to model time-series data in animal vocalizations [36]. These models were particularly effective in capturing temporal patterns in bird songs.
2010s: Deep Learning models viz., CNNs became widely used for audio spectrogram analysis, leading to more accurate classification of animal sounds, including poultry vocalizations. Models such as ResNet and VGG were adapted for sound classification tasks [37]. Recurrent Neural Networks (RNNs) and Long Short-Term Memory (LSTM) were used to capture temporal dependencies in sound sequences, enhancing the recognition of dynamic patterns in animal vocalizations.
2020s: Emergence of TinyML and Edge AI have seen the rise of resource-efficient AI models tailored for deployment on edge devices, particularly in real-time scenarios. Tiny ML, which enables machine learning on low-power microcontrollers, has been pivotal in developing real-time poultry vocalization classification systems. Development boards like Syntiant^™^ [38] with Neural Decision Processor (NDP)along with Key word spotting have been used to deploy lightweight models directly on farms, minimizing latency and power consumption.

Research Gap:

Despite the fact Machine Learning and Deep Learning has been used for animal monitoring, existing approaches often struggle with deployment in real farm settings due to environmental noise, memory constraints, low power and scalability issues. Moreover, there is no specific work carried out to the best of the authors regarding the specific use of Tiny ML for real-time monitoring of chicken vocalizations. Hence the proposed work is carried out with following objectives to close these gaps and improve farm management techniques and chicken welfare by using TinyML for continuous, real-time vocalization monitoring:

- Investigate ways to continually and real-time monitor flocks of chickens using TinyML on low-power edge devices. This include fixing problems with CPU speed, memory size, and battery life.
- Examine strategies for creating large datasets that include a wide range of chicken vocalizations captured in various settings in order to accurately classify animal sounds by distinguishing between minuscule differences in chicken vocalizations using Tiny ML.
- Analyze how the accuracy of vocalization analysis is affected by environmental noise fluctuation.

The principal Contributions of this work are:

- Developed a new, huge dataset containing a variety of chicken vocalizations recorded in a range of physiological and environmental conditions.
- Developed a TinyML application to track chicken vocalizations in real time.
- Created and tested noise-resistant TinyML algorithms that can classify and recognize certain sounds produced by hens with high accuracy.

The remainder of the paper is structured as follows: The materials and methods are described in Section II. Section III discusses the results. Section IV presents the future work folled by conclusions in Section V.

## II Materials and methods

### 2.1 Ethical approval and study location

The study was conducted in accordance with the Declaration of Helsinki and was approved by the Institutional Review Board (IRB) of King Faisal University, Saudi Arabia (Approval No. KFU-REC-2024-MAR-ETHICS2060). The research was conducted in accordance with the laws and rules governing the ethics of studying living things. The experimental trial was carried out at the Avian Research Center, situated approximately 16 kilometers from the King Faisal University (KFU) campus. This facility, located at Guaiba village (latitude 25.383609, longitude 49.584826), houses a dedicated indoor poultry farm for research.

### 2.2 Experimental design for data collection

**Fig 1:**
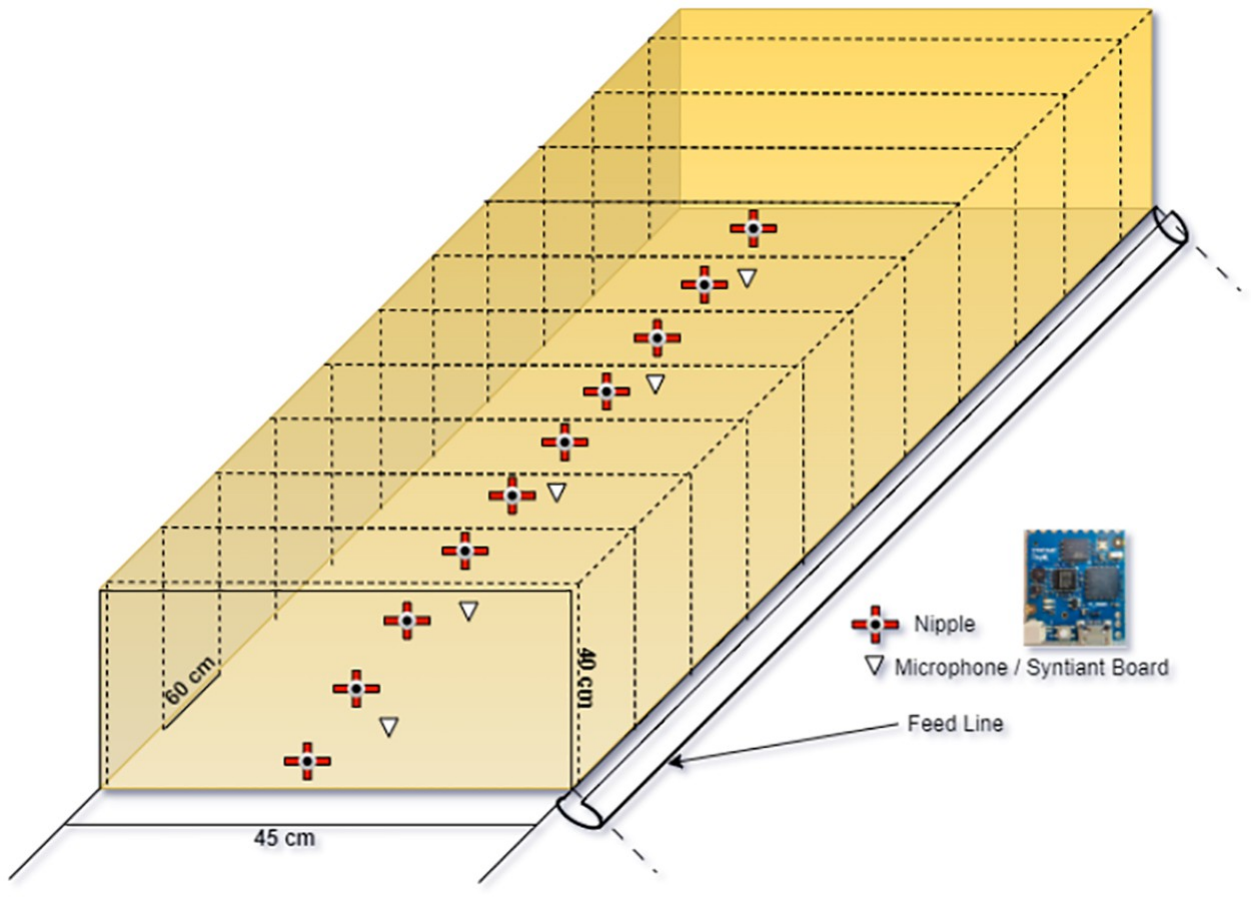
Cage design for experimental data collection.

To record vocalizations, we selected 500 Lohman White hens at 30 weeks of age, sourced from a KFU-affiliated farm at Agriculture Research Station, poultry research unit. In an experimental aviary system, the hens were split into ten pens at random (with 50 hens per pen, 10 cages in each pen, and 5 hens per cage). The size of the room (barn) housing the hens measures 30 meters in length by 20 meters in width by 3 meters in height. A part of the barn included four racks, each holding about 100 chickens. The barn was separated into several sections. Within the barn, there were four compartments of this kind that made it easier to record vocalizations in a protected setting. The hens were kept in a normal lighting setup with a well-ventilated barn that maintained a constant temperature of 21 degrees Celsius with a relative humidity of between 50% and 55% to ensure consistency. To enough air circulation, two sizable ventilation fans were mounted on the barn walls. Each bird was placed in cages with pan feeders and a nipple drinking system, providing hens with adequate feed and water supply throughout the study period. Poultry feed is fed in the feeder tray at 5 AM (120 grams of feed per hen). The measurements of each layer hen in the rack containing the hens (cages) are as follows: each layer is 24 inches (60 cm) long, 18 inches (45 cm) wide, and 16 inches (40 cm) high. Fig 1 displays the precise configuration of these cages inside the racks, mounted with sensors to record vocalizations. The cages were built with sturdy materials to suit the barn’s environmental conditions. The floors of the cages were made with slopes to help collect trash and eggs. trash was allowed to fall through to areas below, while eggs were guided into trays.

To record vocalizations, we used the Syntiant Tiny ML board, which features an ultra-low-power Neural Decision Processor (NDP101) optimized for continuous, on-device audio processing. The device, with an embedded high-sensitivity microphone, was mounted strategically at four locations within the poultry pens to maximize sound capture quality (Fig 1). Data was stored on an SD (Synchronous Dynamic) memory card within the TinyML board, supporting extensive recording without data loss.

### 2.3 Data collection protocol

Recording Schedule: From January 2024 to June 2024, vocalizations were constantly recorded every day for a duration of six months. The following window periods were recorded for 60 minutes each day: 5:00 AM—6:00 AM, 7:00 AM—8:00 AM, 1 PM—2 PM, 5:00 PM—6 PM, 10 PM– 11 PM, and 1 AM—2 AM. These time frames were selected to cover peak activity periods, as well as vocalizations from egg-laying hens such as contentment clucks, hunger piping, alarm/distress calls, exploratory peeping, prelaying crackles, and crowing of rooster.

Recognizing particular sound patterns linked to various behavioral, physiological, and environmental states is necessary to assess the health of chickens based on their vocalizations. Each audio sample was labeled according to the specific behavioral context observed, such as feeding or pre-laying behaviors, as confirmed by avian researchers. Avian experts indicate that the following vocalizations are typical of healthy chickens:

**Table:** 

Types of Vocalization	Characteristics	Indication
Contentment Clucks	Mellow, steady clucking noises	Usually, these noises are connected to feelings of warmth and wellbeing. They show that the chickens are content and at ease in their surroundings
Alarm/Distress calls	Repetitive calls and loud, harsh, high-pitched vocalization	Pain, discomfort, or fear are frequently indicated by distress calls. If the birds are in distress because of predators, physical discomfort, or unfavourable environmental circumstances, you may hear their sounds.
Hunger Piping	High-pitched, repeated peeping or chirping.	The piping of hunger denotes the presence of hunger and the need for food in the fowl. This is a common observation in juvenile birds and chicks
Exploratory Peeping	Quizzical, quiet peeping noises	These sounds are characteristic of engaged and inquisitive birds that are investigating their surroundings.
Prelaying Crackles	Succession of noises produced prior to the laying of an egg	These noises, which are frequently connected to nesting behaviour, signal that a hen is ready to lay an egg.
Crowing of rooster	Loud, crowing sounds	Roosters naturally mark their territory with their crowing, which can also be seen as a sign of vigilance or a desire to mate.

Through the analysis of these vocalizations, one can learn more about the health and welfare of chickens and take timely action to solve any difficulties that may arise.

### 2.4 AI model development

**Fig 2:**
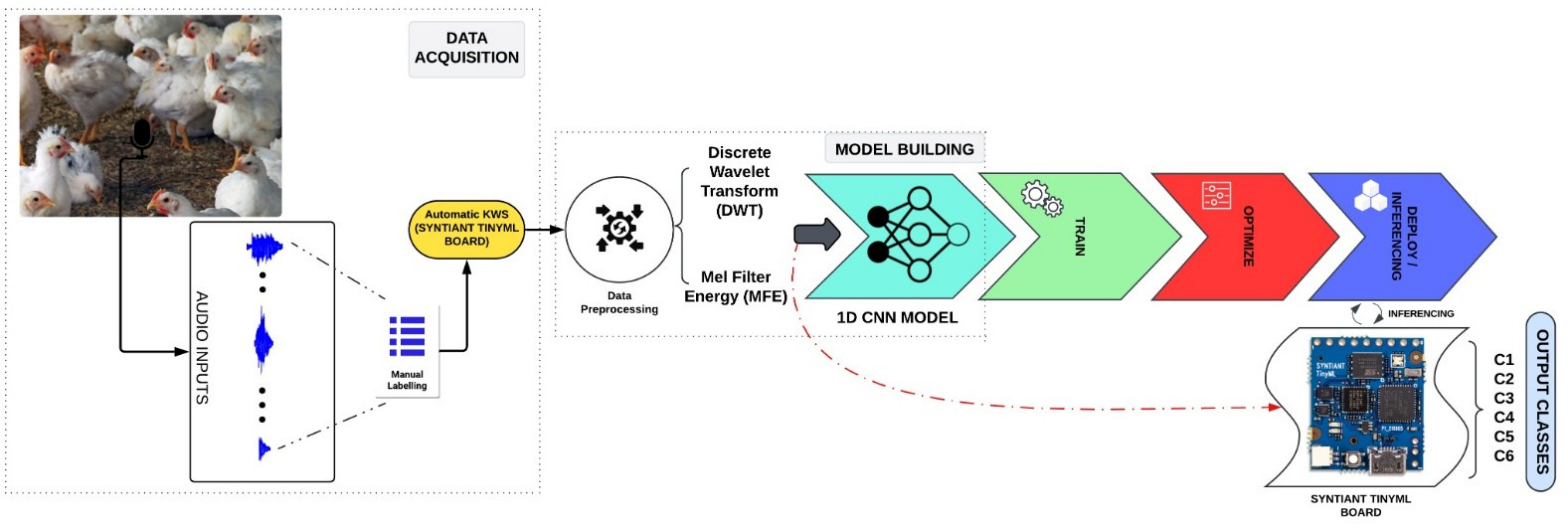
Architecture of neural network processor for poultry vocalization classification [32].

**Fig 3:**
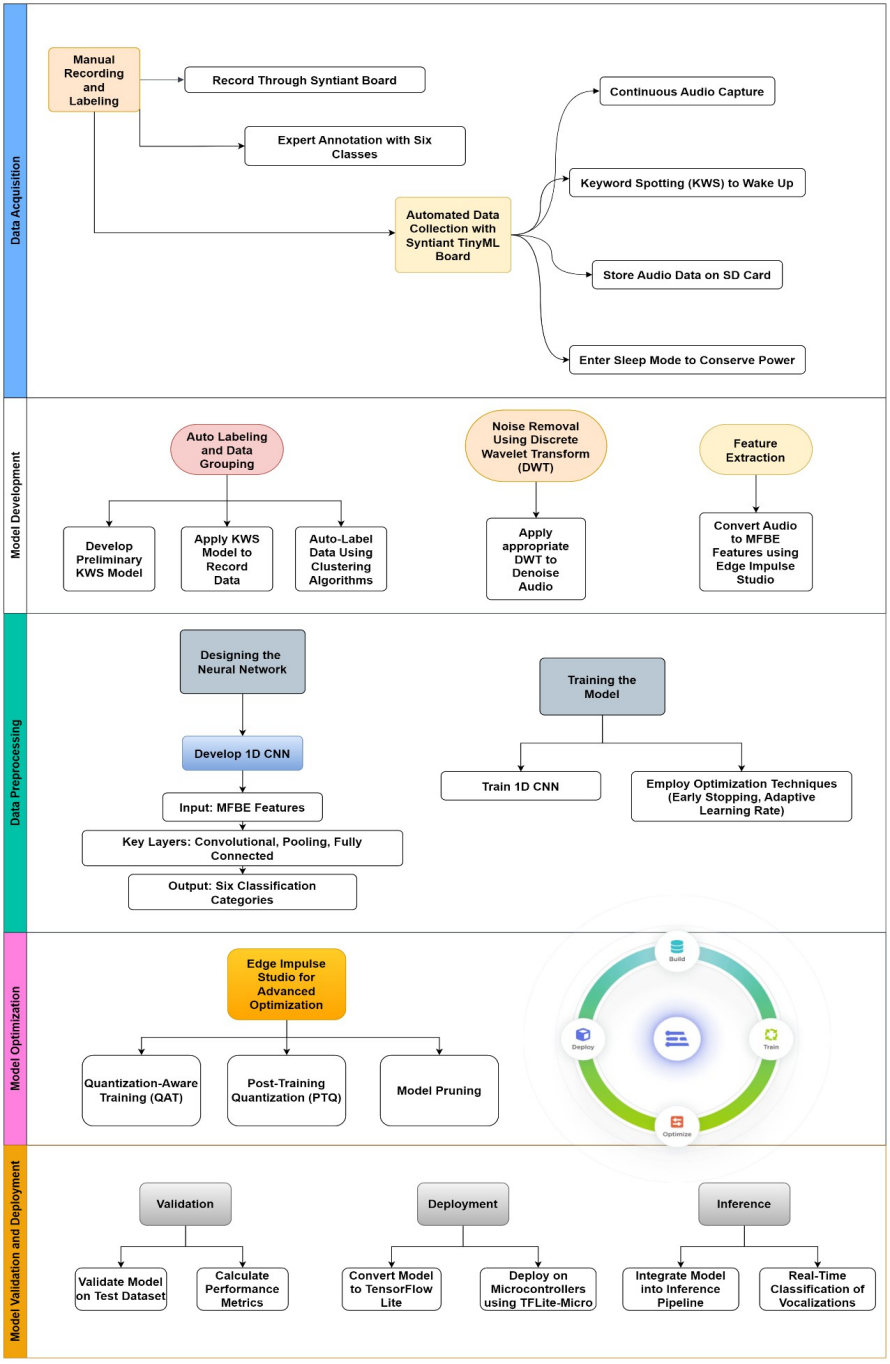
Flow diagram of Tiny ML framework for the proposed architecture.

Fig 2 shows the proposed architecture of Neural Network processor for poultry vocalization classification that integrates various components, blocks to process and classify audio signals very effectively. Fig 3 shows the flow diagram that explains the Tiny ML framework adopted by the architecture for experimentation. Edge Impulse studio [31,32] is the reference for experimental design.

The flow begins with Data Acquisition, featuring manual recording and annotation alongside automated data collection using the Syntiant TinyML board. The data collection process involves continuous audio capture and keyword spotting (KWS) for wake-up functionality, storing audio on an SD card, and entering sleep mode for power efficiency. The Data Preprocessing phase follows, where noise removal is performed using Discrete Wavelet Transform (DWT), and Mel Frequency Band Energy (MFBE) features are extracted using Edge Impulse Studio. Next, Model Development involves designing and training a 1D Convolutional Neural Network (CNN) to classify vocalizations into six categories. The model was trained and validated on an 80–20 training-validation split, achieving a high F1 score across various vocalization classes. Optimization techniques like early stopping and adaptive learning rates are applied. In the Model Optimization phase, advanced techniques such as Quantization-Aware Training (QAT), Post-Training Quantization (PTQ), and model pruning are utilized for deployment readiness. Finally, Model Validation and Deployment ensure the model’s performance on test data, followed by conversion to Tensor Flow Lite and deployment on microcontrollers using TFLite-Micro for real-time inference and classification of poultry sounds.

For statistical evaluation of model performance, we used Python’s SciPy and scikit-learn libraries. These tools enabled the calculation of precision, recall, F1 scores, and confusion matrices to validate model accuracy in classifying distinct poultry vocalizations.

## III Results and discussions

This study used a combination of deep learning techniques, clustering algorithms, and hand labeling to classify chicken vocalizations into six different classes. Contentment clucks (C1), Hunger piping (C2), Prelaying crackles (C4), Rooster crowing (C5), and Alarm/Distress sounds (C6) were the classes that were taken into consideration. Mel Filter Bank Energy (MFE) feature extraction and a one-dimensional Convolutional Neural Network (1D CNN) were used in the classification process. Noise was removed during preprocessing using the Discrete Wavelet Transform (DWT).

**Fig 4:**
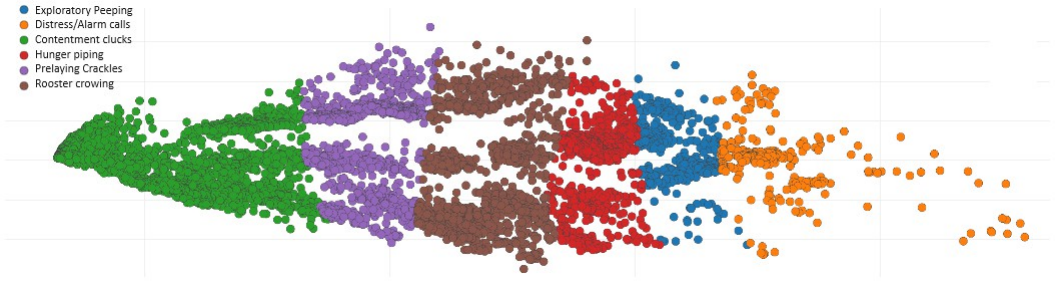
Data for fowl vocalization that has been labeled or annotated for modelling.

The dataset used to model the vocalizations of chickens is displayed in Fig 4. Working with avian specialists, the data was painstakingly classified and annotated. Edge Impulse was used for automatic labeling. The dataset sizes for the following classes are as follows: 1200 contentment clucks; 480 hunger piping; 360 exploratory peeping; 720 prelaying crackles; 600 cock crowing; and 240 alarm/distress sounds. For each class, the datasets were split into training and validation groups at a 4:1 ratio. As recommended by Edge Impulse Cloud Studio, each time series data is divided into segments lasting two seconds to achieve the optimal outcome. For the machine learning methods used to be effective, the model must be trained on high-quality, precisely identified data, which is ensured by this meticulous labeling process.

**Fig 5:**
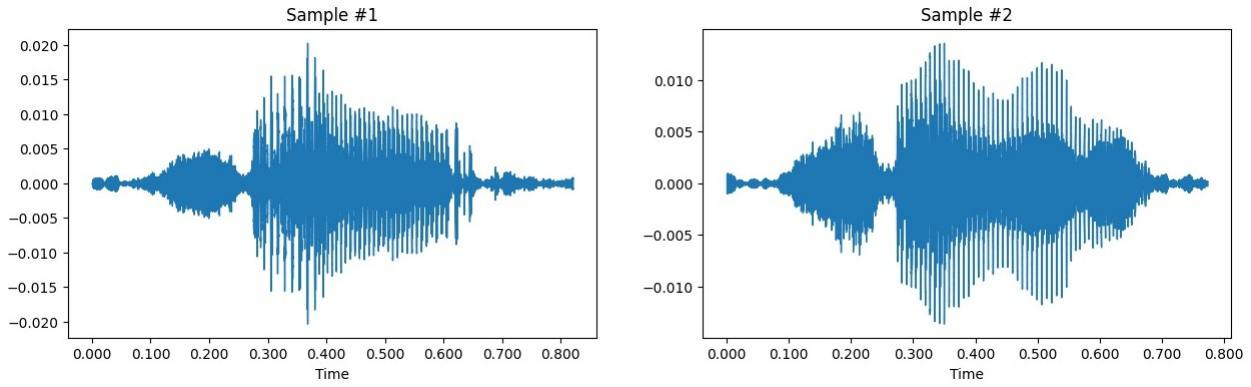
Time series data of two chicken vocalization (Sample #1,#2).

The variety and intricacy of chicken vocalizations are evident in raw audio recordings, as illustrated in Fig 5, which shows time series data for two different chicken vocalizations. These recordings serve as the foundation for further preprocessing stages like feature extraction using Mel Filter Bank Energy and noise reduction with Discrete Wavelet Transform (DWT). This preprocessing is essential, as distinguishing between various vocalization classes can be challenging, highlighting the need for advanced classification methods.

**Fig 6:**
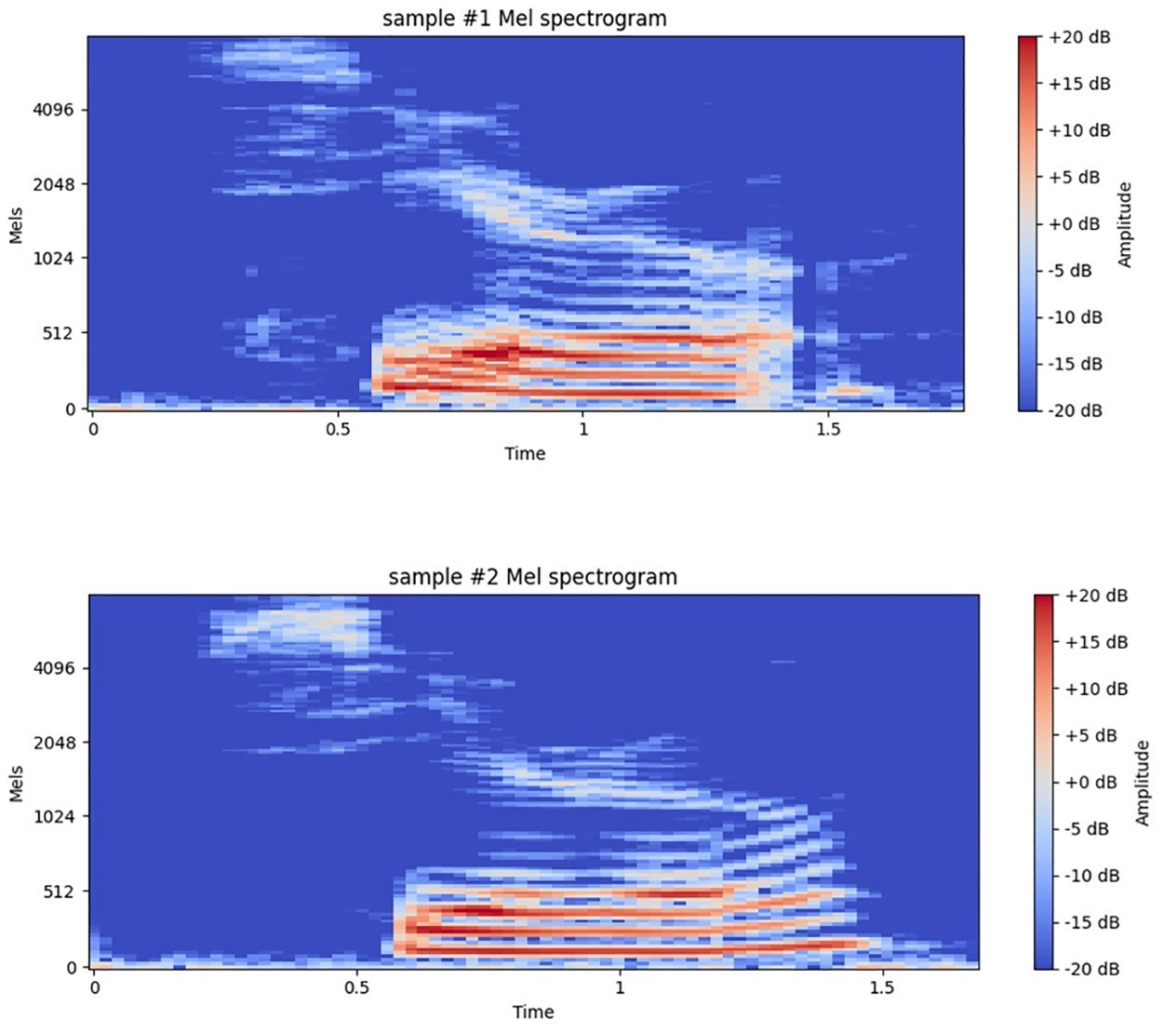
Mel spectrogram for two chicken voice samples (Sample #1, Sample #2).

Mel Spectrograms, as shown in Fig 6, provide a time-frequency domain view of the vocalization data, displaying frequency intensities over time for the two chicken voice samples. This transformation is valuable for classification as it captures important features that help the neural network recognize patterns across vocalizations. By preprocessing the raw audio spectrum of poultry vocalizations with Mel-filter bank energy, the audio data is converted into a representation that aligns better with poultry auditory perception. This approach improves our 1D CNN model’s effectiveness in identifying distinct vocalization classes.

The Mel-filter bank, arranged on a Mel scale, reflects the human auditory system’s sensitivity by assigning a perceptual pitch scale with equal pitch distances.

$$m=2595\;{\text{log}}_{10}\left(1+\frac{f}{100}\right)$$

where ‘m’ is the Mel frequency and ‘f’ is the frequency in Hertz (Hz).

The Mel-filter bank energy calculation involves segmenting audio into overlapping frames through Short-Time Fourier Transform (STFT), applying triangular filters on the Mel scale, and using logarithmic compression to match human loudness perception. For each frame, the Fourier Transform is computed to obtain the power spectrum of the signal.

$$X\left[k\right]=\sum_{n=0}^{N-1}\;x\left[n\right]\cdot e^{-j2\pi kn/N}$$

where X[k] is the k-th frequency component, x[n] is the time-domain signal, and N is the number of samples in a frame. The m-th Mel filter’s energy can be computed using the equation below,

$$E_{m}=\sum_{k=f_{m-1}}^{f_{m+1}}\;{\left|X\left[k\right]\right|}^{2}\cdot \;H_{m}\left[k\right]$$

where Em is the energy in the m-th filter, ∣X[k]∣^2^ is the power spectrum at frequency bin k, and Hm[k] is the response of the m-th Mel filter at frequency bin k.

To mimic the non-linear perception of loudness in the human auditory system, the Mel-filter bank energies are subjected to logarithmic compression as follows,

$$\text{log}\;E_{m}=\text{log}\left(E_{m}\right)$$

where Log Em is the logarithm of the energy in the m-th Mel filter.

By combining Mel-filter bank energy and DWT, we enhance our model’s ability to differentiate vocalizations by minimizing noise and focusing on relevant signal properties. The Discrete Wavelet Transform (DWT) divides a signal into localized wavelets, capturing both frequency and temporal information. This decomposition is achieved by successively applying high-pass and low-pass filters, allowing extraction of detailed frequency information at various scales. The process generates detail coefficients (representing high-frequency components) and approximation coefficients (representing low-frequency components), which are mathematically expressed as follows:

$$a_{j+1}\left[n\right]=\underset{k}{\sum }\;h\left[k-2n\right]a_{j}\left[k\right]$$

$$d_{j+1}\left[n\right]=\underset{k}{\sum }\;g\left[k-2n\right]a_{j}\left[k\right]$$

where aj+1[n] and dj+1[n] are the approximation and detail coefficients at scale j+1, respectively, h[k] is the low-pass filter, and g[k] is the high-pass filter. aj[k] represents the approximation coefficients at scale j.

In our preprocessing pipeline, we applied the Daubechies wavelet (db1) to denoise raw audio signals before feeding them into the 1D CNN for poultry voice classification. Reducing non-stationary noise enhances the input data quality, thus improving the model’s classification performance.

Wavelet denoising is a robust signal processing technique that isolates a signal’s individual frequency components. The main steps are:

Decomposition: DWT divides the audio signal into layers of detail coefficients and approximation levels.

**Fig 7:**
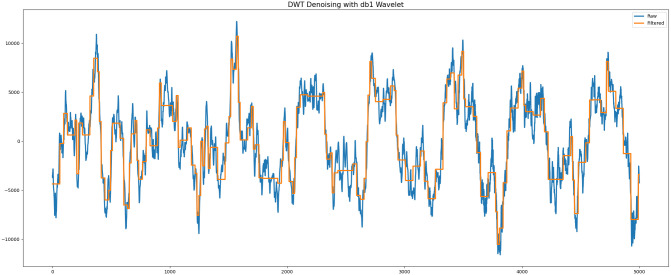
DWT denoising with db1 wavelet for raw chicken vocalization.

Thresholding: To remove noise, the detail coefficients are subjected to a threshold, using either hard or soft thresholding to discard coefficients below a certain value. Fig 7 shows the db1 wavelet-denoised signal, demonstrating how it reduces high-frequency noise while preserving essential vocalization features. This denoising process improves input data quality, optimizing the classification model’s performance.

**Fig 8:**
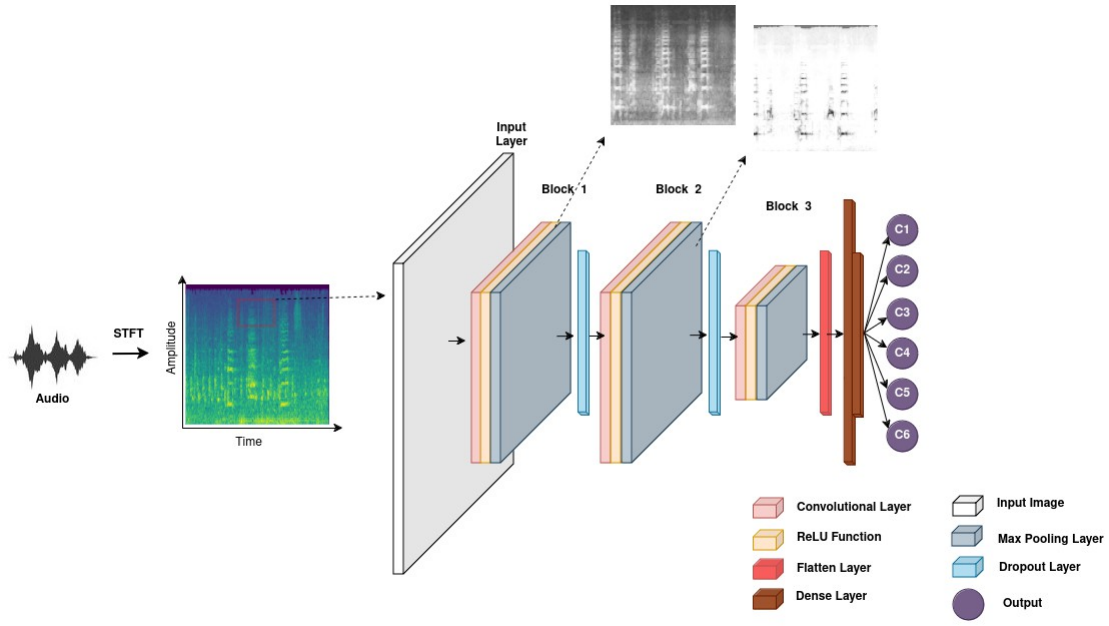
Architecture of 1 D CNN model used for implementation.

The CNN’s performance benefits significantly from DWT and Mel-filter bank preprocessing, as these methods enhance the signal-to-noise ratio, ensuring a cleaner audio input. This preprocessing allows the CNN to recognize distinctive characteristics of poultry vocalizations more accurately, leading to improved classification. The Mel-filter bank energy further reduces data dimensionality, focusing on perceptually relevant information and making the model robust for TinyML implementations. Our 1D Convolutional Neural Network (CNN) architecture is optimized for real-time classification of poultry vocalizations, even in resource-constrained environments. The architecture, depicted in Fig 8, includes three convolutional blocks with Conv1D, MaxPooling1D, and Dropout layers, organized to capture temporal audio patterns efficiently. The CNN uses 32, 64, and 128 filters in successive Conv1D layers to detect sequential patterns, supported by MaxPooling1D and Dropout layers to prevent overfitting. Following the convolutional blocks, a Flatten layer converts multi-dimensional outputs into a 1D vector, suitable for dense layers that complete the classification. Dense layers use ReLu activation, while the output layer employs a sigmoid activation for binary classification. This CNN model effectively combines feature extraction, regularization, and pattern recognition to classify chicken vocalizations accurately. Now the implemented model is evaluated for performances such as Precision, Recall, F1 score. The confusion matrix is a central tool for evaluating a classification model’s performance. This square matrix, typically constructed for multiple classes, summarizes the model’s predictions by showing actual classes in rows and predicted classes in columns. Diagonal elements represent correctly classified instances, while off-diagonal elements indicate misclassified ones.

Performance Metrics

The model’s overall accuracy, or correctness, is calculated as the ratio of correctly predicted instances to the total number of instances.

- Precision (P) measures the proportion of true positive predictions out of all positive predictions, indicating how well the model avoids false positives. High precision is particularly valuable in scenarios where false positives are costly.
- Recall (R), also known as the True Positive Rate or Sensitivity, is the proportion of correctly predicted positive instances out of all actual positives. High recall reflects the model’s ability to identify all relevant instances of a class.
- F1 Score is the harmonic mean of precision and recall, offering a balanced measure when class distributions are uneven. It’s calculated as: F1 = 2PR / (P + R)
- False Positive Rate (FPR) is the ratio of incorrectly predicted positive instances to all actual negative instances. A low FPR is desirable to reduce false alarms.

Together, these metrics provide a comprehensive view of the model’s strengths and weaknesses in classifying data accurately.

The Tables 1 and 2 include performance measures and confusion matrices for two different ways of identifying fowl vocalizations: Mel-filter energy bank and Mel-filter energy bank with denoising using Discrete Wavelet Transform (DWT). The use of DWT denoising improved the overall classification accuracy from 91.67% to 96.11%. This demonstrates the significant role of noise removal in enhancing the model’s ability to correctly classify poultry vocalizations. Similarly, with DWT denoising, precision and recall metrics also show an increase. The precision improved from 91.72% to 96.25%, reflecting a better capability of the model to predict relevant vocalizations. Also, recall rose from 91.67% to 96.11%, indicating a higher rate of correctly identifying true positive instances across all six classes. The F1-Score, which balances precision and recall, also shows a marked improvement (from 91.66% to 96.13%). This highlights the robustness of the classification model after incorporating DWT denoising. DWT effectively removes non-stationary noise components from the audio recordings while retaining the critical features essential for classification. This preprocessing step significantly enhances feature quality, leading to more accurate and reliable classification results.

**Table 1:** Confusion matrix and performance metrics of poultry vocalization classification using mel- filter energy bank.

Class	C1	C2	C3	C4	C5	C6
C1	1087	36	42	21	10	4
C2	27	409	18	23	1	2
C3	22	3	320	1	13	1
C4	18	8	23	650	14	7
C5	32	12	18	8	527	3
C6	21	0	5	0	17	197
**Metrics**						
F1 Score	0.903	0.863	0.814	0.914	0.892	0.868
Precision	0.901	0.874	0.751	0.925	0.905	0.921
Recall	0.906	0.852	0.889	0.903	0.878	0.821
FPR	0.05	0.019	0.033	0.018	0.018	0.05

**Table 2:** Confusion matrix and performance metrics of poultry vocalization classification using mel- filter energy bank with DWT denoising.

Class	C1	C2	C3	C4	C5	C6
C1	1111	17	22	31	9	10
C2	19	431	16	7	5	3
C3	15	1	334	1	9	0
C4	13	9	15	682	1	0
C5	22	12	14	1	549	2
C6	14	0	4	3	4	215
**Metrics**						
F1 Score	0.930	0.907	0.848	0.944	0.915	0.905
Precision	0.929	0.919	0.870	0.944	0.956	0.905
Recall	0.931	0.896	0.827	0.944	0.915	0.905
FPR	0.07	0.015	0.018	0.02	0.011	0.009

The Edge Impulse platform [32] was used to optimize the model through quantization and pruning techniques to prepare it for deployment on resource-constrained devices. Quantization reduces the model’s weight precision, lowering memory and power requirements with minimal impact on accuracy. Pruning removes unnecessary connections within the network, creating a sparser and more compute-efficient model. Thanks to quantization-aware training, which simulates quantization effects during model training, high accuracy is preserved in the final quantized model.

Compact and Scalable Design

- Small Form Factor: The processor’s compact design allows it to be easily integrated into small devices and sensors, making it ideal for deployment in poultry farms.
- Scalability: Its scalable architecture supports deployment in both small and large monitoring systems, catering to various farm sizes and needs.

**Fig 9:**
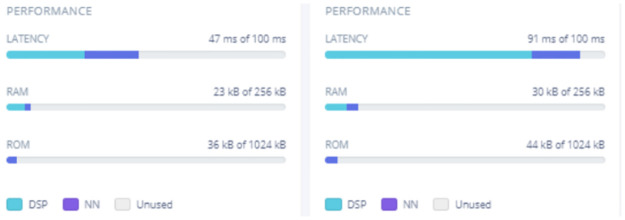
Hardware resource usage targeted to Syntiant TinyML board.

Fig 9 displays hardware resource utilization on the Syntiant TinyML board during inference for both best-case and worst-case scenarios across validation data. The best-case inference latency is 47 ms, demonstrating a swift response time crucial for real-time applications. With 23 kB of RAM used out of 256 kB, ample RAM remains for additional processes or enhancements. The ROM usage is 36 kB out of 1 MB, leaving space for further code or features. In the worst-case scenario, the latency reaches 91 ms, still within acceptable limits for real-time processing, while RAM and ROM usage remain well within available limits. The latency, RAM, and ROM metrics across scenarios indicate that the model is memory-efficient and responsive, supporting future expansion and new features without sacrificing performance. Additionally, the efficient use of hardware resources allows for model scalability and adaptation to more complex tasks or added classifications without significant hardware upgrades, making it suitable for real-time on-device poultry vocalization analysis. The Syntiant TinyML board is optimized for power-efficient edge computing, ideal for battery-operated, always-on devices. With neural networks designed for low-power use, the board performs complex tasks locally, reducing the need for cloud communication. This on-device processing lowers energy consumption related to data transmission and latency, and its architecture supports efficient inference operations, extending battery life while maintaining high performance. This feature set makes it a strong choice for speech recognition, audio processing, and sensor data analysis in constrained environments.

**Fig 10:**
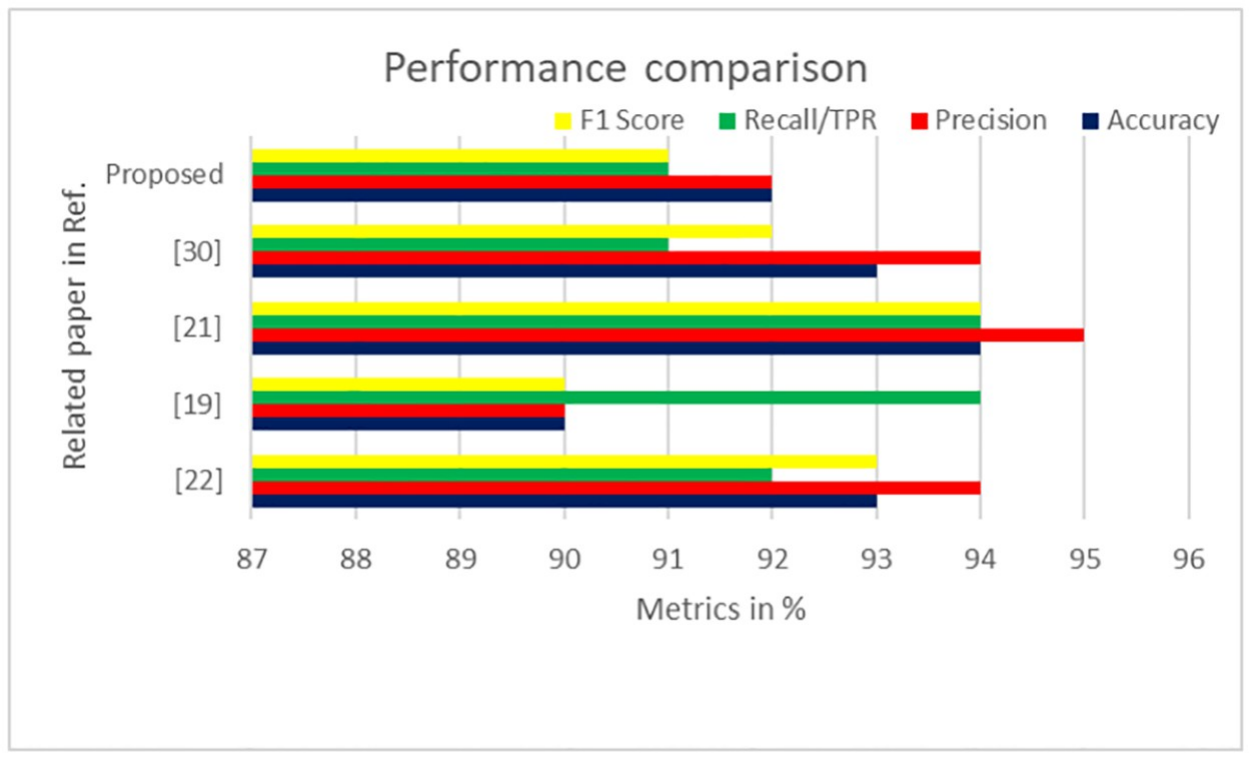
Performance metrics comparison with related works.

Comparison with Related Work:

Fig 10 presents a performance comparison between the proposed model and related works across various metrics.

- [22] uses MFCC for distress classification, achieving high accuracy (93%), precision (94%), recall (92%), and F1 score (93%). However, it focuses solely on distress detection, limiting its utility across a broader range of vocalizations.
- [19] applies MFCC, MFE, spectral features, and wavelet entropy, with the highest recall (94%) using wavelet entropy. Although it has good sensitivity, its lower accuracy (90%) and precision (90%) suggest potential issues with false positives or negatives.
- [21] employs deep learning models like ResNet, VGG11, and Light-VGG11, achieving the best results with Light-VGG11 (accuracy 94%, precision 95%, recall 94%, F1 score 94%). However, this approach requires more RAM and incurs higher latency, making it less suitable for resource-constrained applications.
- [30] uses various classifiers (SVM, DT, Naïve Bayes, Random Forest, CNN, and KNN) for poultry vocalization classification. KNN achieves the best results, though the overall approach is computationally intensive, slow, and memory-demanding, posing challenges for large datasets and real-time applications.

The proposed model integrates multiple feature extraction and optimization methods, including DWT for noise reduction and Mel Filter Bank Energy for feature extraction, similar to [19,22]. It achieves an accuracy of 92%, precision of 92%, recall of 91%, and an F1 score of 91%. While these metrics are slightly lower than the best-performing models ([21,22]), the proposed model’s use of "int8" quantization ensures a lightweight design deployable on a 32-bit microcontroller with 256 kB of RAM and 1 MB of flash memory. This balanced performance profile makes the model viable for real-world applications, especially where resources are limited, and diverse vocalization types need accurate classification.

Limitations:

1. Variability in Vocalizations: Even among members of the same class, poultry vocalizations can vary significantly. The acoustic characteristics of the sounds might vary depending on factors including age, breed, and individual differences. Due to its inherent unpredictability, the model may not always be able to distinguish between vocalizations that sound identical, which could produce mediocre results.
2. Noise and Overlapping Sounds: Real-world audio data frequently contains overlapping sounds and background noise, which can be challenging to completely remove, even when Discrete Wavelet Transform (DWT) was utilized for noise removal. The model’s capacity to recognize and categorize the target vocalizations accurately may be hampered by these background noises.
3. Complexity of the Model: The classification was performed using a one-dimensional convolutional neural network (1D CNN). Though efficient, more sophisticated or customized designs, like transformers or recurrent neural networks (RNNs), might be able to better capture the complex patterns and temporal connections in the vocalization data.

## IV Future work

Prospective investigations can concentrate on improving the accuracy of the model by investigating sophisticated methods for feature extraction and classification, in addition to tackling its constraints. Further refining the model to accommodate a wider variety of poultry habitats can be achieved by augmenting the dataset with additional annotated instances and vocalization classes. To increase the model’s resilience and conditional adaptability, methods like data augmentation and transfer learning might be investigated.

## V Conclusions

In conclusion, real-time analysis of chicken vocalizations can be achieved with the help of the suggested 1D CNN architecture since it successfully strikes a balance between efficiency and performance. Within the limitations of edge computing technology, this architecture can accurately classify poultry vocalizations by utilizing spectral components, convolutional feature extraction, and model optimization techniques. This method not only improves the well-being of chickens by keeping an eye on their health constantly, but it also shows off TinyML’s potential for use in agriculture. With major ramifications for the poultry industry, the study’s encouraging overall findings open the door to the development of complex, automated systems for the analysis of chicken vocalizations.

## Supporting information

S1 Dataset (ZIP)
